# Discordance in diagnosis of osteoporosis using spine and hip bone densitometry

**DOI:** 10.1186/1472-6823-5-3

**Published:** 2005-03-11

**Authors:** Alireza Moayyeri, Akbar Soltani, Nasibeh Khaleghnejad Tabari, Mohsen Sadatsafavi, Arash Hossein-neghad, Bagher Larijani

**Affiliations:** 1 Endocrinology & Metabolism Research Centre, Shariati Hospital, Tehran University of Medical Sciences, Tehran, Iran; 2 Research Development Center, Evidence Based Medicine Working Team, Shariati hospital, Tehran University of Medical Sciences, Tehran, Iran

## Abstract

**Background:**

Diagnostic discordance for osteoporosis is the observation that the T-score of an individual patient varies from one key measurement site to another, falling into two different diagnostic categories identified by the World Health Organization (WHO) classification system. This study was conducted to evaluate the presence and risk factors for this phenomenon in a large sample of Iranian population.

**Methods:**

Demographic data, anthropometric measurements, and risk factors for osteoporosis were derived from a database on 4229 patients referred to a community-based outpatient osteoporosis testing center from 2000 to 2003. Dual-energy X-ray absorptiometry (DXA) was performed on L1–L4 lumbar spine and total hip for all cases. Minor discordance was defined as present when the difference between two sites was no more than one WHO diagnostic class. Major discordance was present when one site is osteoporotic and the other is normal. Subjects with incomplete data were excluded.

**Results:**

In 4188 participants (3848 female, mean age 53.4 ± 11.8 years), major discordance, minor discordance, and concordance of T-scores were seen in 2.7%, 38.9% and 58.3%, respectively. In multivariate logistic regression analysis, older age, menopause, obesity, and belated menopause were recognized as risk factors and hormone replacement therapy as a protective factor against T-score discordance.

**Conclusion:**

The high prevalence of T-score discordance may lead to problems in interpretation of the densitometry results for some patients. This phenomenon should be regarded as a real and prevalent finding and physicians should develop a particular strategy approaching to these patients.

## Background

Osteoporosis is defined as a systemic skeletal disease characterized by low bone mass and micro-architectural deterioration of bone tissue, with a consequent increase in bone fragility and susceptibility to fracture [1,2]. This definition indicates that measurement of bone mineral density (BMD) is a central component to diagnosis of the disease [3].

'T score' is a statistical definition which indicates the difference between patient's BMD and mean bone density of normal population in the age of 20 – 30 (reference population) [3]. This value shows the difference in terms of standard deviations. According to the World Health Organization (WHO) classification system, T scores under the value of -2.5 are considered as osteoporosis and between -1 and -2.5 as osteopenia. These figures are usually calculated separately for two different sites of lumbar spine and total hip.

Discordance in diagnosis of osteoporosis is defined as presence of different categories of T scores (osteoporosis, osteopenia, and normal) in two skeletal sites of an individual patient [4]. This phenomenon has been divided into two groups: major and minor [5]. Minor discordance happens when the different diagnostic classes are adjacent; i.e., patient is diagnosed as osteoporotic in one site and osteopenic in the other site, or, osteopenic in one site and normal in the other site. If the diagnosis is osteoporosis in one site and the other site is in the normal range, the discordance falls into the major class.

Actually, one of the reasons for measuring BMD in several sites is the presence of discordance, which can affect the diagnosis and therapeutic plan in an individual person. Various studies have analyzed the prevalence and impact of T-score discordance on different aspects of management of osteoporosis [5-9]. However, most of these studies did not evaluate risk factors for this phenomenon.

Given this background and concerning the need for the estimation of the impact of this phenomenon in our country, we aimed to evaluate the presence and risk factors for T-score discordance in a large sample of Iranian population.

## Methods

Participants in this study were 4229 persons who underwent bone densitometry in outpatient clinic of Endocrinology & Metabolism Research Center in Tehran from 2000 to 2003. A considerable proportion of these cases were healthy post-menopausal women referred by clinicians for densitometric evaluations. All study participants signed the informed consent for any scientific approach to their medical registered data. Our Institutional Review Board approved this study.

A standardized questionnaire was filled before densitometry for all participants. Demographic data (including age and sex) as well as other known or suspicious risk factors for osteoporosis (including menopause, age at menopause, age at menarche, history of osteoporotic fractures, drugs, and smoking) were collected. All participants had their standing height measured using a stadiometer to the nearest 0.5 cm. Weight was measured on a standard weighting scale with a precision of 0.5 kg. Body mass index (BMI) was calculated as weight (kg) divided by height (m) squared. All the BMD measurements were done for diagnostic purposes and none of the participants were on the treatment with bone active agents (hormone replacement therapy was not considered a bone active agent).

BMD was measured at the lumbar spine and total hip with dual X-ray absorptiometry (DXA) using a Lunar DPXMD densitometer (Lunar 7164, GE, Madison, WI) by a trained operator according to the manufacturer's instruction. The instrument was calibrated weekly by using appropriate phantoms. Precision error for BMD measurements was 1–1.5% in the lumbar and 2–3% in the femoral regions. The device normative data of US population for spine BMD and NHANES III study for femur BMD were used as reference values.

All the data gained from densitometry and questionnaires were entered into a comprehensive relational database. The participants with incomplete data were excluded from the study. To compare presence of various risk factors in participants with and without T-score discordance, chi-square test and independent sample t-test were used firstly. Potential risk factors were entered to a multivariate binary logistic regression analysis and the resulted odds ratios with 95% confidence intervals were reported. *P *values less than 0.05 were taken to indicate statistical significance. Statistical analyses were performed using Stata Statistical Package, version 8.0 (Stata Corporation, College Station, Tx).

## Results

In sum, 4188 persons were enrolled in the study. Characteristics of all participants are summarized in Table 1. The main reasons of referral for BMD measurement were menopause in 49%, old age in 16%, glucocorticoid use in 9%, history of low energy fractures in 1.5%, and other reasons (such as metabolic disorders, rheumatoid arthritis, positive family history, leanness, and transplantation) in 4.5% of participants. In 20% of participants, no major risk factor was identified as the referral reason.

**Table 1:** Characteristics of the study population*

	**Male participants (n = 340)**	**Female participants (n = 3848)**
**Age (years)**	49.7 (16.3)	53.8 (11.2)
**Weight (kilograms)**	68.5 (13.1)	67.1 (11.9)
**Height (centimeters)**	168.5 (7.7)	156.1 (6.1)
**Body Mass Index (kg/cm^2^)**	24.1 (4.2)	27.6 (4.7)
**History of osteoporotic fracture**	8 (2.4)	47(1.2)
**Smoking**	35 (10.3)	94 (2.4)
**Corticosteroid use**	89 (26.2)	298 (7.7)
**Hormone Replacement Therapy**		231 (6.0)
**Age at menarche (years)**		13.6 (1.5)
**Menopause**		2137 (55.5)
**Age at menopause (years)**		47.2 (5.8)
**Femoral T score**	-0.93 (1.24)	-1.43 (1.18)
**Lumbar T score**	-1.40 (1.48)	-1.45 (1.54)

* Numbers are presented as mean (standard deviation in parenthesis) for numerical variables and frequency (percentage in parenthesis) for categorical variables.

Totally, 518 participants were diagnosed in osteoporotic range in hip area and 1036 participants in the lumbar area. T-score classifications are presented in Table 2. Major discordance was observed in BMD results of 115 (2.7%) participants. Minor discordance was observed in 1631 (38.9%) participants and T-score categories of two measurement sites in other 2442 (58.3%) participants were not different. Distribution and pattern of this variable in different genders is depicted in Table 3.

**Table 2:** Classification of T scores according to WHO criteria in different sites*

	**Lumbar spine**			**Total hip**		
	*No.*	*%*	*95% Confidence Intervals*	*No.*	*%*	*95% Confidence Intervals*

**Osteoporosis (T = -2.5)**	1036	24.7	23.4–26.0	518	12.4	11.4–13.4
**Osteopenia (-2.5 < T = -1)**	1605	38.3	36.8–39.8	1592	38.0	36.5–39.5
**Normal (T > -1)**	1547	36.9	35.5–38.4	2078	49.6	48.1–51.1

**Table 3:** Distribution of diagnostic discordances according to WHO criteria in different genders*

	Male participants (n = 340)	Female participants (n = 3848)	Total (n = 4188)
**Major T-score Discordance**	**7 (2.1)**	**108 (2.8)**	**115 (2.7)**
Hip Osteoporosis, Normal Lumbar	5	16	21
Hip Normal, Lumbar Osteoporosis	2	92	94
**Minor T-score Discordance**	**117 (34.4)**	**1514 (39.3)**	**1631 (38.9)**
Hip Osteoporosis, Lumbar Osteopenia	10	99	109
Hip Osteopenia, Lumbar Osteoporosis	39	515	554
Hip Osteopenia, Normal Lumbar	35	220	255
Hip Normal, Lumbar Osteopenia	33	680	713
**T-score Concordance**	**216 (63.5)**	**2226 (57.8)**	**2442 (58.3)**
Hip and Lumbar Osteoporosis	50	338	388
Hip and Lumbar Osteopenia	93	690	783
Hip and Lumbar Normal	73	1198	1271

* Numbers are presented as frequency (percentage in parenthesis).

T-score discordance was more prevalent in women than men (42.2% versus 36.5%, P = 0.042). The mean age of participants with discordance (54.8 years) was higher than the other group (52.5 years, P < 0.001). In 3848 female participants, the number of post-menopausal women with diagnostic discordances (951 of 2027) was significantly higher than pre-menopausal participants with discordance (671 of 1821; P < 0.001). In multivariate analysis (Table 4), two genders lost their difference in occurrence of discordance. Effects of age and menopause were established with their significant odds ratios. Participants with late menopause (age at menopause > 50) were more likely to show T-score discordances. Obesity defined as BMI over 30 was recognized as a risk factor for major discordance and smoking as a protective factor against minor discordance. Hormone replacement therapy was a significant protector against both.

**Table 4:** Results of multivariate logistic regression analysis for risk factors of major and minor discordance getting T-score concordance at lumbar and femoral sites as the reference

**Variables**	**Minor Discordance**	**Major Discordance**
**Gender (female)**	1.09 (0.85 – 1.4)	1.02 (0.45 – 2.3)
**Age decade**	1.2 (1.1 – 1.3)*	1.5 (1.2 – 1.9)*
**Age group (>65 years)**	1.2 (1.01 – 1.6)*	1.4 (0.70 – 2.7)
**Corticosteroid use**	0.89 (0.73 – 1.1)	0.71 (0.37 – 1.3)
**Body Mass Index (>30 kg/cm^2^)**	1.01 (0.87 – 1.2)	1.7 (1.2 – 2.6)*
**History of osteoporotic fracture**	1.1 (0.59 – 2.0)	1.3 (0.29 – 5.5)
**Smoking**	0.66 (0.45 – 0.97)*	0.49 (0.12 – 2.1)
**Menopause**	1.3 (1.1 – 1.5)*	1.7 (1.01 – 2.7)*
**Hormone Replacement Therapy**	0.37 (0.16 – 0.82)*	0.54 (0.36 – 0.82)*
**Age at menarche (> 13 years)**	1.1 (0.90 – 1.3)	0.82 (0.50 – 1.3)
**Age at menopause (> 50 years)**	1.4 (1.1 – 1.7)*	2.0 (1.2 – 3.4)*

* indicates significant odds ratio. Numbers are presented as odds ratio (95% confidence intervals in parentheses).

## Discussion

This study reveals that, using WHO criteria for definition of osteoporosis and osteopenia, a significant fraction of patients (41.7% in this study) would show T-score discordance between hip and spine sites. Most of these discordances (38.9%) are from minor category, presenting difference on only one class, and could be due to minor variation in BMD techniques or some minor physiologic dissimilarity. Minor discordance generally does not influence the overall prognosis of patients; however, in the case of patients with one site normal and the other osteopenic, follow up of patients with hip osteopenia seems reasonable [7].

The multivariate analysis we have implemented to the data could aid clinicians and diagnosticians to approach patients with different characteristics. According to our results, BMD measurement in both sites is necessary at least for older patients and post-menopausal women especially those with delay in menopause. Hormone replacement therapy, however, could decrease the diagnostic discordance and patients receiving estrogen and progesterone are more likely to have similar results in DXA scans of lumbar and femoral areas. This could be the result of drug effects on the BMD of lumber area [10].

Generally, five different causes have been proposed for occurrence of discordance [5]. Physiologic discordance is related to the skeleton's natural adaptive reaction to normal external and internal factors and forces. An example of this type of discordance is the difference observed between the dominant and non-dominant total hip. Pathophysiologic discordance is seen secondary to a disease. Common examples include vertebral osteophytosis, vertebral end plate and facet sclerosis, osteochondrosis, and aortic calcification. Anatomic discordance is owing to differences in the composition of bone envelopes tested. An example is the difference in T-scores found for the PA lumbar spine and the supine lateral lumbar spine in the same patient. Artifactual discordance occurs when dense synthetic substances (such as metal from zipper, coin, clip, etc) are within the field of region of interest of the test. And finally, technical discordance occurs when the technician improperly positions the patient for the test or the hardware or software used to acquire the test data is out of order.

Major discordance was observed in 2.7% of our participants, which is in agreement with the results of similar studies. In both major and minor discordances, lower BMD for lumbar spine was more prevalent. This could be due to several reasons. The difference between velocities of bone loss in different parts of human body could be the main reason [11]. Trabecular bones (typical of lumbar area) are known to have a more rapid rate of deprivation in early post-menopausal state in comparison to cortical bone (typical of proximal femur) [12]. Moreover, most of the etiologies of the secondary osteoporosis (such as glucocorticoid excess, hyperthyroidism, malabsorption, liver disease, rheumatoid arthritis, and medications) first affect spinal column [13]. This will lead to higher prevalence of lumbar osteoporosis. In addition, weight bearing is a known cause of physiologic dissimilarity, which can cause rise in bone density especially in the hip and femur regions [14]. This mechanism could be the reason of more major T-score discordances observed by increment of BMI in this study.

In 30% of our participants, the lumbar T-score was higher than hip T-score and this culminated in poorer hip diagnoses in 9.2% of participants. This phenomenon could be regarded as 'inverse discordance' and several factors may be involved in its occurrence. One of these reasons is the prevalent vitamin D deficiency in our participants. A recent nationwide study with random sampling from five major cities in Iran reported a high prevalence (about 80%) for vitamin D deficiency in Iranian population [15]. Other studies have confirmed this finding [16,17]. Basic studies have revealed that decrease in serum concentrations of vitamin D by means of raising serum parathyroid hormone (PTH) would induce reduction in density of cortical bones and may have a supportive role for density of trabecular bones [18]. The other reason for 'inverse discordance' could be due to other diseases such as minor compression fractures in lumbar area, joint sclerosis, and aortic calcification [19,20]. These ailments can induce errors in the estimation of lumbar BMD and falsely higher values.

The observation of 'inverse discordance' could not be regarded as a direct influence of more significant bone loss in femoral region. A known phenomenon named 'birth cohort effect' can play a role [21]. This indicates that, in the particular section the data have been gathered, a specific observed finding could not be interpreted for the effects of age and time passing. In this study, the reason for lower femoral BMD can be insufficient bone gain during puberty in this area. Latest findings indicates that peak bone mass of Iranian population are about 5% lower than that of western population [22,23]. Decreased bone density in hip region could lead to start of bone loss from lower amounts in older ages and post-menopausal states. This can lead to femoral osteoporosis without significant decrease in lumbar BMD.

This study, as every other cross-sectional study, has a number of limitations. We could not rule out the possibility of referral bias for this study. As the study was performed in a referral center affiliated to a teaching hospital, the assumption of similarity of study population to exact community is not reasonable and we could not generalize the results to the Iranian population. The other limitation is the choice of multivariate analysis used in this study. With the current analysis, prediction of the presence or absence of T-score discordances is possible. However, prediction of the situation of one site according to results of the other site or choosing one site to measure BMD need further evaluations and analyses which was behind the scope of this study. Future studies using more powerful statistical analyses with larger sample sizes are needed to establish these imperative questions.

The importance of existing discordance on the prognosis and fracture risk of patients needs further prognostic studies with long follow-up designs. The high prevalence of T-score discordance could induce some problems for the physicians in decision-making regarding these patients. In general, high prevalence of discordance in this study and similar studies suggests some defects in the cut-off values for definition of osteoporosis and osteopenia proposed with the WHO [5]. To eliminate this problem, further studies to re-calculate ranges for definition of these diagnoses (considering diagnostic and therapeutic necessities) seem to be needed.

## Conclusion

In summary, this study indicates that about 40% of participants evaluated for bone density changes in a referral center may show diagnostic discordance, majority of them from minor class. This phenomenon should be regarded as a real and prevalent finding and physicians should become familiar with this topic. Clinicians should look for possible cause or causes of this occurrence and develop a particular strategy approaching to these patients.

## Competing interests

This study was supported by a grant from Endocrinology & Metabolism Research Center of Tehran University of Medical Sciences.

## Authors' contributions

In advance, suggestion of the design of the study was from AS. Data extraction and initial analysis were done by NKT and AH. AM performed additional analyses and wrote the first draft of the paper. AS and BL both had helpful and valuable comments in revising the paper. All authors read and approved the final manuscript.

## Pre-publication history

The pre-publication history for this paper can be accessed here:


